# Macrophages in Intestinal Wound Healing: Dichotomous Effects and Therapeutic Opportunities

**DOI:** 10.3390/ijms27104508

**Published:** 2026-05-18

**Authors:** Alexander D. Bungert, Maximiliane Merle Winter, Andreas Pascher, Felix Becker

**Affiliations:** 1 Department of General, Visceral and Transplant Surgery, University Hospital Muenster, 48149 Muenster, Germany; andreas.pascher@ukmuenster.de (A.P.); felix.becker@ukmuenster.de (F.B.); 2 Department of Gynecology and Obstetrics, University Hospital Düsseldorf, 40225 Düsseldorf, Germany; maximilianemerle.winter@med.uni-duesseldorf.de

**Keywords:** anastomotic leakage, macrophage polarization, wound healing, immunomodulation

## Abstract

Anastomotic leakage (AL) is a significant complication associated with elevated morbidity and mortality rates following colorectal surgery. This complication primarily arises due to impaired wound healing. Anastomotic and intestinal wound healing is generally divided into three phases: inflammation, proliferation, and remodeling. The physiological transition between these phases is primarily orchestrated by macrophages, which are key regulators of inflammation and tissue repair. They undergo sequential phenotypic changes from pro-inflammatory to anti-inflammatory states and are involved in the phagocytosis of bacteria or debris, but also attract fibroblasts for collagen production and deposition. Importantly, they can promote local perfusion by secreting pro-angiogenic and growth factors. Failure of this transition from pro- to anti-inflammatory properties is associated with AL, scarring, and fibrosis. Intestinal macrophages represent the largest pool of resident myeloid cells and are promising cellular targets for therapeutic interventions. In this narrative review, we focus on intestinal and anastomotic wound healing, highlight the dichotomous role of macrophages, and discuss potential therapeutic strategies. A detailed understanding of macrophage polarization, recruitment, and targeted modulation may enhance wound healing and prevent complications such as AL.

## 1. Introduction

Intestinal wound healing is traditionally divided into three phases: inflammation, proliferation, and remodeling [1]. As the gastrointestinal tract contains the largest pool of macrophages in the body, these cells are key players in regulating intestinal wound healing and are abundant at all phases [2,3]. The inflammatory cascade is initiated upon injury or surgical procedure. Activation of the innate immune system leads to the proliferation of resident immune cells that phagocytose debris and bacteria [4,5]. During the early inflammatory phase, macrophages exhibit pro-inflammatory M1-like phenotypes that later transform into pro-healing phenotypes during the proliferative phase. Ultimately, the complex mechanism of intestinal wound healing terminates with the remodeling phase, in which macrophages possess fibrolytic properties for extracellular matrix (ECM) remodeling [6,7]. Macrophages undergo specific phenotypic changes and exert different functions at each phase [8,9]. Disturbances in this tightly regulated process can cause fibrosis, scarring [3] or anastomotic leakage (AL). Anastomotic leakage is one of the most feared complications of intestinal surgery and results from impaired healing, leading to a loss of tissue integrity. Due to leakage of luminal content, there is a high risk of abdominal sepsis or death [10]. It affects 1–28% of patients who undergo gastrointestinal surgery, significantly increases morbidity and mortality, and represents a large economic burden [11,12,13,14]. This review highlights the current state of knowledge regarding the dichotomous role of macrophages in intestinal wound and anastomotic healing (AH), as well as therapeutic options. This article is a narrative review; literature was searched broadly without a formal systematic search protocol or PRISMA-compliant study selection process.

## 2. Materials and Methods

This article is a narrative review. No systematic search protocol, PRISMA flow diagram, or predefined study selection criteria were applied. Literature searches were conducted in PubMed, Google Scholar, and the Cochrane Library using the following search terms in varying combinations: “macrophage”, “macrophage polarization”, “M1 macrophage”, “M2 macrophage”, “intestinal wound healing”, “anastomotic healing”, “anastomotic leakage”, “colorectal surgery”, “intestinal fibrosis”, “mucosal healing”, and “inflammatory bowel disease”. No language or date restrictions were applied; however, preference was given to articles published within the last ten years. Reviews, original research articles, and translational studies in humans, rodent models, and *in vitro* systems were considered. Studies focusing exclusively on non-intestinal wound healing without relevance to general or intestinal macrophage biology were excluded.

## 3. Intestinal Wound Healing

**Figure 1:**
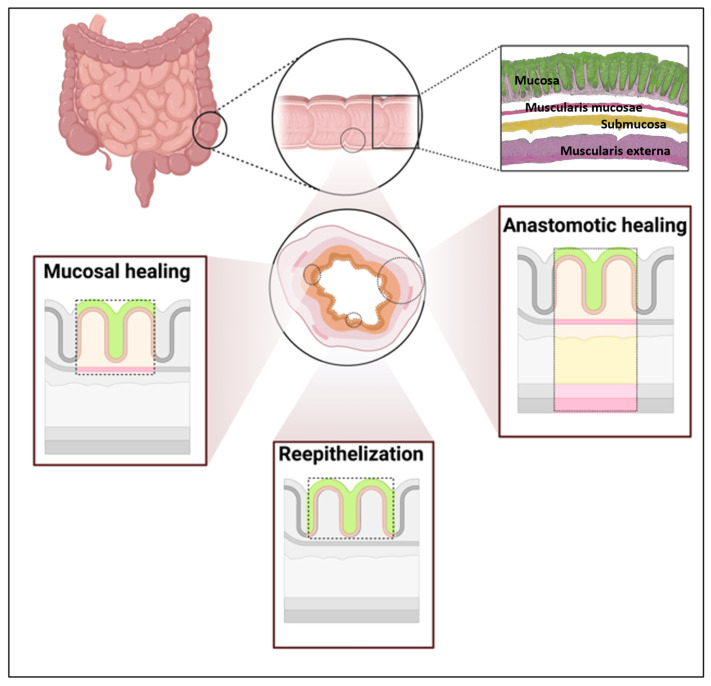
Intestinal wound healing occurs in different layers of the small and large bowel. It is subdivided into the re-epithelization process for superficial wound healing and mucosal or AH for deeper tissue defects. The upper-right inset shows the anatomical layers of the intestinal wall, including the epithelial/mucosal surface, submucosa and muscularis externa. In the schematic healing panels, these layers are simplified (from the inside out): green highlights the regenerating epithelial/mucosal surface, pink represents the underlying mucosal compartment with lamina propria including the muscularis mucosae in dark pink, pale yellow indicates the submucosa, light pink represents the external muscular layer and dark pink the outer boundary and adjacent tissue. Created in BioRender.

Intestinal wound healing encompasses superficial re-epithelialization (epithelial restitution), mucosal wound healing of deeper defects (e.g., ulcers in inflammatory bowel disease (IBD)), and anastomotic healing following reconstruction. The small and large intestines consist of four layers: mucosa, submucosa, muscularis propria or externa, and serosa. The innermost layer of the alimentary tract is the intestinal epithelium, which represents the largest mucosal surface in the body and separates the lumen and its microbiota from the mucosal immune cells [15] (Figure 1). In the case of injury, the first stage of response is hemostasis, which prevents excessive blood loss and seals the tissue. Following hemostasis, an inflammatory cascade is initiated [16]. In response to necrotic or damaged cells, damage-associated molecular patterns (DAMPs) are released. DAMPs include DNA, histones, high-mobility group protein (HMGP), and adenosine triphosphate (ATP). They induce inflammation in a feedback loop by recognizing pattern recognition receptors (PRRs) on surrounding neutrophils [17,18]. Neutrophils are the first immune cells to appear in large numbers after injury. Murine experiments on skin wounds have shown that recruitment begins 4–6 h after injury and peaks 18–24 h after onset [19] shaping the immunological prerequisites for monocyte recruitment due to pathogen clearance and debris removal. Macrophages are mainly recruited as monocytes via monocyte chemoattractant protein (MCP-1/CCL2) after 24–48 h and differentiate into macrophages once they reach their destination [20,21]. They emerge as the central orchestrators of wound healing, which will be discussed in the following sections.

### 3.1. Superficial Re-Epithelization

The epithelium is continuously renewed and, following injury, undergoes restitution via transition to a migratory phenotype before redifferentiating to restore tight junctions [22,23,24]. Following injury and coagulation, Seno *et al.* found that clotted material was no longer present and was substituted by a monolayer of epithelial cells. This is managed by the transition of normally polarized enterocytes to a squamoid appearance [23,25]. Subsequently, redifferentiation to the normal phenotype is initiated and finally terminated with the formation of tight junctions between adjacent epithelial cells [23]. Resident and recruited macrophages contribute to this process by sensing epithelial damage and releasing mediators, such as TGF-β, IL-10, prostaglandins, and growth factors, that promote epithelial proliferation and wound closure [26,27].

### 3.2. Mucosal Healing

Mucosal healing refers to the repair of deeper mucosal defects and is a critical endpoint in the management of IBD. In IBD, genetic, microbiological, or environmental factors lead to persistent epithelial dysfunction. As soon as the mucosal barrier is broken, translocation of antigens to the subjacent lamina propria occurs, leading to sustained mucosal inflammation and defective resolution [28,29]. Consequently, effective mucosal healing is closely associated with durable remission, reduced hospitalization, and decreased need for surgery. In a healthy state, inflammation is limited and begins with the influx of DAMP-activated neutrophils. After migration across the epithelium, neutrophil-derived proteases cleave intestinal epithelial junctions, and the cleaved products lead to increased proliferation of the surrounding epithelial cells. The release of chemokines, including CCL20 and CCL2, facilitates the influx of blood-derived monocytes, which then differentiate into macrophages [20,30]. Macrophages and neutrophils produce pro-resolution (lipoxins, resolvins, protectins) and proliferation factors, such as VEGF, nitric oxide, and TGFβ, and therefore support the resolution of inflammation [16,31]. Macrophages are subsequently polarized to M2-like macrophages to clear apoptotic neutrophils [16].

### 3.3. Anastomotic Healing (AH)

AH is a distinct form of intestinal wound healing characterized by the surgical tissue approximation of two tubular structures, such as intestinal organs, exposure to luminal microbiota, and mechanical stress. During surgery, reconnection of the gastrointestinal passageway after tissue resection is an essential step, followed by AH, a particularly complex form of intestinal wound healing. It involves several tightly orchestrated processes performed by local and recruited immune cells to rebuild the tissue continuum and restore the functional architecture exhibiting the three well-known overlapping phases of general wound healing [32,33]. The submucosa represents the layer with the highest tensile strength and is therefore considered decisive for successful wound healing. It mainly consists of collagen and elastin fibers [34]. Intestinal AH should be regarded as a separate field of research, considering the numerous factors that impact the physiology and pathology of the digestive tract [35]. There is clear evidence that intestinal AH differs significantly from cutaneous healing [36]. In contrast to cutaneous healing, the gastrointestinal tract consists of three different types of collagen (I, III, and V), whereas in cutaneous healing, only collagen I and III are of primary importance [34]. In addition, collagen synthesis is regulated differently [37]. Second, the conditions in the gastrointestinal tract are entirely different from those in the skin. Shear stress is present from the outset due to the passage of food and peristaltic waves. In addition, the flora of anaerobic and aerobic bacteria with constant contamination can influence AH, with aerobic bacteria rarely causing problems in skin injury. The tensile strength of the new anastomosis decreases during the inflammatory phase due to lysis of the former collagen within the first three days after surgery. Thereafter, the newly formed collagen increases the strength to approximately 75% of the preoperative strength in the colon, but never reaches the initial level. In small bowel anastomoses, full recovery of wound strength can be achieved four weeks after surgery [34,37]. The biological distinctness of the gastrointestinal tract (specifically, reliance on type V collagen and its constant exposure to microbiota) creates a healing environment in which any dysfunction of macrophages directly transitions into AL [38]. It is assumed that an impaired phenotypic switch from pro-inflammatory (M1) to pro-resolving (M2) macrophages causes (prolonged) wound inflammation, which then affects AH. Decreased growth factor secretion is associated with altered wound closure, vascular networks, and collagen composition [39].

### 3.4. Anastomotic Leakage (AL)

AL is one of the most feared complications following bowel resection and is associated with postoperative morbidity (e.g., sepsis) and mortality rates of up to 39%, as well as high additional financial costs for the healthcare system [40,41,42]. In a review of 97 studies, 56 different definitions of AL were used. However, AL is usually defined as any clinical sign of leakage, regardless of whether it was diagnosed clinically, radiologically, or endoscopically [43]. For example, the incidence of AL after colorectal cancer surgery varies between 3% and 12%, depending on tumor location, surgical approach, and patient characteristics, such as age, sex, comorbidity, and lifestyle. Several risk factors are responsible for AL, including smoking, alcohol abuse, obesity, and diabetes [44]. Mechanistically, growing evidence suggests that the intestinal microbiota modulates macrophage polarization, thereby either driving persistent inflammation and barrier failure or supporting adequate anastomotic wound closure [45]. Another factor that can be partially influenced by surgery is tissue perfusion. Anatomical circumstances must ensure adequate local tissue perfusion; however, sufficient cardiac output with good arterial tissue oxygen pressure is also decisive. Hypovolemia leads to malperfusion of the gastrointestinal tract, thereby limiting oxygen delivery to the anastomotic site, preventing the initiation of collagen synthesis, and leading to wound healing disorders [34]. In the first postoperative days, the integrity of the tissue of newly created anastomoses relies mainly on technical issues; however, later AL is mostly caused by immunological effects [46].

In recent years, increased attention has been paid to immunological processes that occur within newly created tissue junctions. The innate immune system plays a crucial role, with macrophages being the predominant cell population. Their functions are described in the following sections.

## 4. Intestinal Macrophages: Origin, Niches, and Phenotypes

### 4.1. Origin

Macrophages are cells of the innate immune system and are part of the mononuclear phagocyte system (MPS). They originate from the bone marrow and are called monocytes as circulating cells that differentiate into tissue-specific macrophages, such as osteoclasts (bone), histiocytes (interstitial connective tissue), and Kupffer cells (liver), and are then referred to as monocyte-derived macrophages [47,48]. Most tissue macrophages are generated before birth, originate from the yolk sac or fetal liver, and remain constant owing to self-renewal and longevity [49]. In mice, intestinal macrophages are mostly replaced by bone marrow-derived Ly6C monocytes in the steady-state intestine [50], where they differentiate into pro-inflammatory M1 or pro-resolving M2-macrophages, depending on cellular and humoral signals [51]. The expression of CCR2 is crucial for the replenishment of the intestinal macrophage pool, as demonstrated by experiments on *Ccr2ko* mice or the lack of CCL2 [49,52,53,54]. Resident macrophages, characterized as MHCII^+^CD11b^+^F4/80^+^ [55], guard physiological gut homeostasis [56] by balancing pathogen tolerance and silent phagocytosis [57] maintaining the integrity of the tissue [58] and epithelial barrier [59] following an inflammatory stimulus.

### 4.2. Niche Model

Different cell compartments, such as the brain, liver, lungs, and intestines, harbor myeloid cells that originate from the bone marrow or are generated prenatally due to influx from the yolk sac or fetal liver, respectively [60,61,62]. In the intestine, depletion of resident macrophages, increased turnover, and epithelial barrier leakage promote peripheral influx and monocyte infiltration into the intestine. The “niche model” describes the communication [63] between the different cell compartments called “niches” and circulating cells. Macrophage engraftment in the intestine occurs only if the niche is open, that is, if there is niche accessibility. This is generally the case for the first phase of inflammation or post-surgery, when endothelial leakiness is increased. The next step is niche availability, which means that there is a need for further cells and an unoccupied niche. If both prerequisites exist, monocytes can engraft and differentiate into cells that adjust to the microenvironmental requirements [64]. Monocytes recruited from the bone marrow can be proinflammatory and can quickly transition into pro-wound-healing or pro-resolving cells. A particular macrophage response may be largely controlled by the type, specific location, and magnitude of the injury, as well as whether it is acute or chronic.

### 4.3. Monocytic and Macrophage Ontogeny and Phenotypes

Monocyte recruitment is initiated by the secretion of chemokines, such as CCL2 and CCL20, by many cell types (e.g., neutrophils, endothelial cells, lamina propria cells, and monocytes themselves) at the site of inflammation after injury of any kind [20,30]. Initially, inflammatory monocytes (CX3CR1^low^CCR2^high^Ly6C^high^ in mice and CD14^++^CD16^−^ in humans) migrate during the early phase of inflammation and represent the major mononuclear cell type. Subsequently, non-classical monocytes (CX3CR1^high^CCR2^low^Ly6C^low^ in mice and CD14^low^CD16^+^ monocytes in humans) follow [16,65]. These monocytes produce different chemokines; the first cell type, the pro-inflammatory one, produces TNFα and IL-6, whereas the latter produces anti-inflammatory cytokines, such as VEGF or TGFβ [65]. Monocytes either die at the site of inflammation through apoptosis [16] or transform into tissue-resident macrophages or dendritic cells (DCs). While Ly6C^high^ monocytes give rise to TNF- or iNOS-producing dendritic cells (Tip-DCs) or inflammatory macrophages, Ly6C^low^ monocytes give rise to alternatively activated macrophages [66].

Based on *in vitro* experiments [67], macrophages are often divided into three phenotypically and functionally distinct categories: M0 naïve/unstimulated macrophages, M1 pro-inflammatory/antimicrobial, and M2 a, b, c, and d: anti-inflammatory/pro-healing/alternatively activated or tumor-associated macrophages. They are functionally distinct from M1 macrophages and can be further subdivided (Table 1), However, this classification oversimplifies the *in vivo* conditions, as it is generally assumed that there is a continuum from M1 to M2 macrophages. *In vivo* wound macrophages behave differently from their *in vitro* counterparts, and their transcriptomes and surface markers often do not correspond between *in vitro* and *in vivo* studies [68,69]. Overall, the phenotypes of macrophages change with wound maturation [70]; therefore, they represent a mixed pool of macrophages with different phenotypes. This is not surprising, as it has already been shown in other organ contexts, such as brain tumors, where the M1/M2 paradigm does not meet the current criteria of knowledge [71,72,73].

### 4.4. Phase-Specific Macrophage Dynamics

#### 4.4.1. Inflammation and Resolution

**Figure 2:**
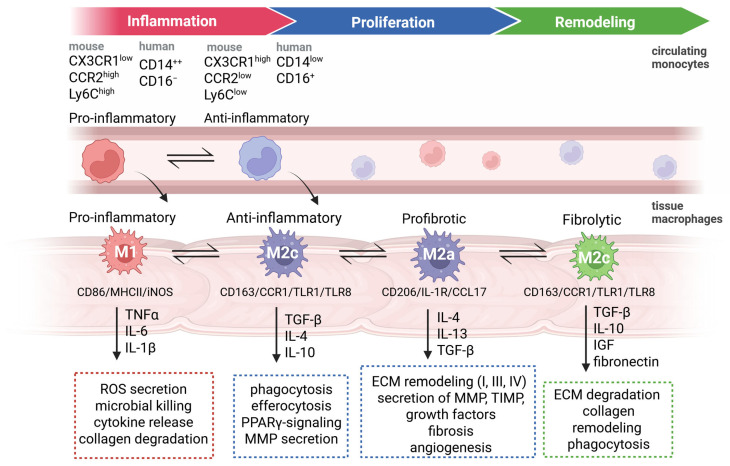
The most important phenotypes, signaling pathways, and cytokines that define the individual transformation steps in the wound healing process are illustrated. Wound healing occurs in three phases: inflammation, proliferation, and remodeling. Macrophages are key players that transform into different phenotypes, each representing different tasks that modulate the transition from inflammation to restoration of the initial tissue injury. Created in BioRender.

The inflammatory phase can be divided into early (1–4 days after wounding) and late (5–7 days after wounding) phases. With the occurrence of proinflammatory M1-like macrophages (CX3CR1^low^CCR2^high^Ly6C^high^ in mice and CD14^++^CD16^−^ in humans), the prevalence of neutrophils decreases by phagocytosis of apoptotic neutrophils and cell debris [65] (Figure 2). In an acute lung injury model, the ingestion of neutrophils also leads to the release of TGF-β and IL-10, which are strong triggers of inflammation resolution and tissue repair [76,77]. Consequently, the influx of neutrophils decreases with the cessation of self-recruitment. The entire process of inflammation-specific phagocytosis, called “efferocytosis” [78,79], is important because ineffective or incomplete efferocytosis can lead to chronic inflammation [80] and persistent tissue-degrading enzymes. Furthermore, high levels of proinflammatory and low levels of pro-repair cytokines would remain [81,82,83]—all conditions that could predispose to AL. Complete resolution of inflammation (i.e., the inflammatory phase) is essential for the restoration of normal tissue function [84]. After completion of this early inflammatory stage, the late inflammatory stage is driven by macrophage phagocytosis of apoptotic cells, which induces the expression of anti-inflammatory cytokines. This process is mediated through several signaling pathways, including PPAR-γ (Peroxisome proliferator-activated receptor γ) activation [85,86]. Consequently, the proinflammatory cell burden diminishes [59]. Apoptosis [87] and differentiation of monocytes into resident macrophages with a non-inflammatory gene expression profile follow [52]. This phase is therefore characterized by a more anti-inflammatory milieu with cessation of TNF-α and IL-1β secretion, but production of MMPs, Il-10, and VEGF [30,86,88,89]. The dominant cell type is the above-mentioned CX3CR1^high^CCR2^low^Ly6C^low^ (in mice) and CD14^low^CD16^+^ (in humans) monocytes, which give rise to M2c-like anti-inflammatory macrophages and resident tissue macrophages (Figure 2).

Finally, it is worth mentioning that all these different stages of inflammation and the prevailing cell types represent a continuum and are partly present at the same time, influenced by external stimuli and cell ontogeny. Moreover, knockout experiments with either *Ccr2ko* or *Cx3cr1ko* mice, which aim to prevent the influx of either pro- or anti-inflammatory monocytes, have shown that wound macrophages persist, revealing the redundancy of macrophages in the affected tissue [90].

#### 4.4.2. Proliferation—Remodeling

Following the inflammatory phase, the remodeling phase initiates wound repair. This process is mainly mediated by profibrotic M2a macrophages via the production of chemokines or growth factors, such as PDGF, TGF-β1, IGF-1, and VEGFα, for cell proliferation and blood vessel development, as well as the synthesis of many ECM components and matrix-remodeling enzymes [1]. Macrophages regulate and secrete matrix metalloproteases (MMPs) and tissue inhibitors of matrix metalloproteinases (TIMPs) for ECM turnover and repress inflammation by efferocytosis and phagocytosis of apoptotic cells [91] (Figure 2). Heparan sulfate is a major component of the ECM and potentiates the effects of VEGF. VEGF, in turn, mediates neo-angiogenesis in the wound bed, resulting in the formation of new capillaries and ensuring the supply of nutrients to the newly formed tissue [92]. VEGF-induced angiogenesis accelerates wound healing and improves anastomotic strength [93]. Macrophage-dependent release of TGF-β not only leads to the resolution of inflammation but also triggers tissue repair by inducing major ECM proteins, such as fibronectin and collagen, promoting ECM, and inhibiting ECM degradation. This mechanism is initiated by TGF-β mediated differentiation of fibroblasts into myofibroblasts [94,95].

Another example of the crucial role of macrophages is macrophage-derived arginase, which converts arginine into ornithine, which serves as a substrate for proline synthesis. This amino acid is the main component of collagen [96].

#### 4.4.3. Remodeling—Reorganization

Finally, the last step of tissue remodeling is maturation, which restores the tissue to its original form. This process involves apoptosis of present cells, including endothelial cells, macrophages, and myofibroblasts, degradation of excessive ECM, and regression of newly formed blood vessels. M2c fibrolytic macrophages play a role in the ingestion of cell debris and degradation of the ECM [92] (Figure 2). Although experiments in a skin tissue model showed that depletion of macrophages in the final remodeling stage did not substantially impact the outcome of the wound healing process, depletion in the inflammatory mid-stage led to severe hemorrhage, indicating the phase-dependent redundancy of macrophages [8]. Nevertheless, Rohani *et al.* found that specific macrophage-derived MMP-10 mediates the expression of macrophage-released MMP-8 and MMP-13, thereby enhancing collagenolytic activity without reducing collagen production by fibroblasts. Overall, *Mmp10ko* mice exhibit stiffer and more collagenous tissue, emphasizing the crucial role of macrophages [97]. Disturbances in these processes lead to scarring and fibrosis, causing fatal consequences in AH. In the context of dermal tissue wound healing, IL-10 deficient mice showed increased macrophage influx and acceleration of wound closure, but impaired biomechanical stability with amplified deposition of collagen, emphasizing the role of IL-10 in scar-free wound healing [98]. Additionally, overexpression of TGF-β in a lung injury model leads to uncontrolled proliferation and differentiation of lung fibroblasts, and therefore aggravation of pulmonary fibrosis [99].

In the intestine, these transitions (inflammation, proliferation, and remodeling) occur within a microenvironment shaped by microbial products, oxygen gradients, and mechanical stress, rendering the balance between pro- and anti-inflammatory activity particularly fragile.

## 5. Evidence from *In Vitro* and *In Vivo* Models: What Do We Know?

**Figure 3:**
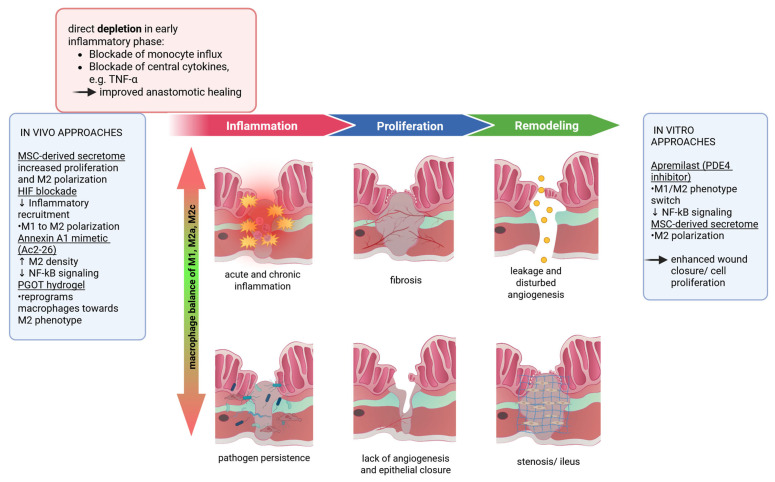
Balanced M1 and M2 macrophage responses are required for normal progression throughout the intestinal wound healing phases. Disturbances in macrophage polarization may lead to persistent inflammation, infection, fibrosis, impaired epithelial repair, or anastomotic leakage. Potential macrophage-targeted therapeutic strategies have been shown [100,101,105,110,111,112]. Created in BioRender.

As macrophages are key players in wound healing and phenotypic changes, such as shifting from M1 to M2 polarization, are critical for tissue regeneration, questions arise as to whether and how these cells can be targeted to enhance wound healing or reduce AL. Hence, predominant strategies have involved depletion or attempts to change the polarization of macrophages to an M2-like phenotype at different time points. The following sections discuss current evidence from *in vitro* and *in vivo* rodent models (Figure 3). To date, robust clinical evidence from randomized controlled trials or large prospective cohort studies is scarce.

### 5.1. In Vitro Models

A review of the current literature reveals a lack of standardized *in vitro* intestinal macrophage models. Only a few *in vitro* approaches have been reported to polarize macrophages and sustainably improve wounds and AH. Mohr *et al.* could show that Apremilast, a phosphodiesterase-4 (PDE4) inhibitor, is able to induce a phenotype switch in human macrophages from M1 to M2 through inhibition of the NF-κB-pathway und downregulation of inflammatory pathways. Moreover, this leads to enhanced fibroblast migration and accelerated wound closure *in vitro* [100]. Yu *et al.* demonstrated that a human umbilical cord mesenchymal stem cell (hucMSC)-derived secretome significantly promoted cell proliferation *in vitro*. Consecutive rodent *in vivo* experiments have shown improved AH mediated by M2 macrophages [101] (Figure 3). However, it should be noted that M2 macrophages may also exert undesirable effects on wound healing. This was demonstrated in a skin-wound mouse model. Ex vivo polarized macrophages showed a clear M2 marker profile *in vitro,* but did not improve or even delayed wound healing after injection into skin wounds of diabetic mice due to the persistence of neutrophils [102].

### 5.2. In Vivo Models

A study by Sengul *et al.* has recently shown that matrix remodeling genes of the mucosa and submucosa are downregulated in defective anastomotic healing in a mouse model. Since bulk RNAseq was the method used, these gene changes could not exclusively be assigned to only macrophages but still show the potential harmful effect of impaired ECM remodeling [103]. *In vivo* studies with respect to only macrophages have reported conflicting results regarding their role in AH. The main obstacles are heterogeneous study designs and the lack of standardized intestinal or AH evaluation tools. Nevertheless, most protocols aim to deplete monocytes and macrophages using various regimes and then compare healing after intestinal manipulation in the presence and absence of macrophages. However, the importance of NOD2, a pattern recognition receptor that is commonly associated with IBD when mutated, has been examined in a study by Witte *et al.* They performed ileocecal resection in wildtype and *Nod2ko* mice, showing that lack of NOD2 leads to impaired wound healing in terms of bursting pressure after 5 days, but a tendency to increased anastomotic leakage with generally lower levels of macrophage-derived inflammatory cytokines [104]. In general, most *in vivo* studies are based on the common understanding that macrophages divide into well-known subpopulations with dichotomous, phase-specific functions: M1 macrophages (iNOS^+^) are thought to have pro-inflammatory effects during early wound healing, and M2 macrophages (CD206^+^) have proliferative and pro-reparative activities. In the case of an imbalance between M1 and M2 macrophages, a sustained M1 proinflammatory status can lead to AL [88]. In a study by our group using gene set enrichment analysis (GSEA), we tracked the genetic effects of macrophage depletion in a mouse model on signaling pathways. Winter *et al.* showed that time-specific depletion of macrophages in the inflammatory phase could dampen the inflammatory response, whereas depletion during the proliferative or remodeling phase did not show any clinical impact on AH but did show differences in gene regulation [105]. Many studies have consistently suggested that pro-inflammatory M1 macrophages aggravate AH and postoperative bowel function, and that temporary depletion or suppression especially in the inflammatory phase could be beneficial (Figure 3). In a recent clinical cohort study with colorectal cancer patients, Hajjar *et al.* showed that increased activity of inflammatory M1 macrophages/monocytes could lead to AL. Preoperatively elevated monocyte counts were more frequently associated with postoperative leakage. Similarly, monocyte depletion prevents AL and abscesses [106]. After surgical manipulation of the abdomen, another study showed that liposome-mediated resident macrophage depletion in a rodent model drastically reduced the secretion of MIP-1α, interleukins IL1β, IL6, ICAM-1, and MCP-1, as well as leukocyte recruitment. In this case, the absence of tissue-resident macrophages also improved intestinal contractility and physiological gastrointestinal transit, indicating that macrophages are responsible for postoperative ileus in patients after surgical procedures [107]. Further studies have emphasized the importance of pro-regenerative M2 macrophages in intestinal anastomotic wound healing. A positive ratio of M2 to M1 macrophages increases the biomechanical strength of anastomoses, which was proven in a rat model [108,109].

Strowitski *et al.* induced murine macrophages to M2 macrophages via blocking the hypoxia-inducible factor and could show ameliorated AH in a septic or ischemic environment. This was reflected in the increased anastomotic bursting pressure and fewer insufficiencies. Mechanistically, this has been attributed to elevated collagen density and diminished recruitment of inflammatory cells by M2 macrophages [110]. Treatment with Collagen-IV-targeted annexin 1 biomimetic peptide Ac2-26 in the early postoperative phase in a murine colitis model undergoing surgery, as reported by Reischl *et al.*, led to increased M2 macrophage density (Figure 3). This correlated with the suppression of the NF-kB signaling cascade and typical proinflammatory mediators, which manifested as better AH and postoperative recovery [111]. While this study administered Ac2-26 intraperitoneally, the same inflammation-resolving nanoparticle was delivered orally in a subsequent mouse study, which showed reduced colitis activity as well as improved anastomotic wound closure postoperatively [112]. In this study, DSS colitis was initiated seven days prior to surgery along with the oral administration of this drug.

### 5.3. Critical Summary of the Macrophage Targeted In Vitro and In Vivo Studies

Several therapeutic strategies targeting macrophage polarization in the context of anastomotic and intestinal wound healing have been reported in preclinical studies. The following structured comparison highlights their mechanisms, evidence base, safety considerations, proposed timing, and clinical feasibility (Table 2).

None of the above strategies has been validated in randomized controlled trials in humans, and most evidence is derived from rodent models, which do not fully recapitulate the human intestinal immune microenvironment. The translational challenge is significant: the timing of macrophage phenotype transitions in humans remains poorly characterized, and reliable intraoperative or perioperative biomarkers (e.g., systemic cytokine profiles, monocytic surface markers) to guide intervention are lacking. Furthermore, the apparent safety of systemic immunomodulation (e.g., with Apremilast) in a perioperative setting warrants prospective evaluation, as modulating macrophage polarization broadly may also impair defense against surgical-site infections.

## 6. Conclusions and Clinical Implications

### 6.1. Summary

In summary, proper intestinal wound healing is strongly dependent on the timely progression of phenotypic changes in macrophages, indicating a correct transformation from M1 (pro-inflammatory) to M2c (anti-inflammatory), M2a (profibrotic macrophages), and finally, M2c (fibrolytic) macrophages (Figure 2). Experimental models in the skin and other tissues have shown that the conversion of M1 macrophages to M2 macrophages is critical [114]. Sustained M1 activation leads to chronic inflammation, impaired and delayed healing [85,115] and an increased likelihood of AL [88]. Conversely, early conversion of M1 to M2 macrophages reduces the phagocytosis of bacteria and debris. However, the microbiome itself can cause intestinal leakage [116]. Furthermore, M2 macrophages are essential for resolution and tissue repair; excessive stimulation and permanent or premature M2 activation (especially profibrotic M2 macrophages) lead to intestinal fibrosis and tissue strictures, especially in IBD [3,117].

The impact of macrophage function and dysfunction on intestinal healing during the later proliferative and remodeling phases, especially the long-term, has not been extensively studied, based on the assumption that early depletion mitigates harmful hyperinflammation, whereas depletion during the later proliferative or remodeling phases is considered to have little effect because of functional redundancy [90]. Most studies have intervened at the beginning of wound healing, that is, during the inflammatory phase. However, it could be advantageous to promote or deplete macrophages in a targeted manner, depending on their phenotypic appearance, analogous to Winter *et al.*, where macrophage depletion in the proliferative phase improved wound healing [105]. However, only a few therapeutic strategies targeting either enhancement of the anti-inflammatory response or suppression of the inflammatory milieu have shown improved AH. A key consideration for macrophage-targeted therapeutic strategies is that the accessibility of the tissue macrophage niche and consequently engraftment of monocyte-derived macrophages is largely confined to the inflammatory phase. As previously reported in Section 5.1, cell-based therapies are used to promote macrophages towards M2 polarization at the anastomotic site, consequently improving wound healing [101]. The same applies to pharmacological interventions that can alter and stimulate macrophages to exhibit reparative M2-like features *in vitro* [100].

Furthermore, newly engineered materials, such as injectable hydrogels (PGOT), directly applied to colonic anastomosis, accelerate healing with reduced AL, accompanied by histological evidence of reprogrammed macrophages to the M2 CD206^+^ phenotype [113]. Beyond cellular targeting, recent data from a mouse model suggest that miRNA-mediated regulation of macrophage polarization toward either M1 or M2 phenotype may offer additional, more precise options in AH [118].

### 6.2. Translational Gap and Future Directions

In summary, the available data do not support a simple dichotomy of “harmful M1” and “beneficial M2” macrophages but instead point to a highly context and phase-dependent role of these cells in anastomotic healing. Integrating advanced *in vivo* models with single-cell and spatial transcriptomic analyses could clarify the timing and mechanisms by which macrophage subsets contribute to healing, fibrosis, or AL. From a clinical perspective, current evidence suggests that macrophages are promising yet highly time-sensitive therapeutic targets in AH. However, reliable biomarkers, such as systemic cytokine profiles or monocytic signatures, are required to stage the current healing phase and should be integrated in future clinical trials. Translating this knowledge into macrophage-targeted therapies may significantly reduce AL and improve surgical outcomes.

## Figures and Tables

**Table 1:** Different macrophage subtypes relevant to wound healing.

Subtype	Induced by…	Marker Expression	Secretion of…	Function	Relevance to Wound Healing
M1	IFNγ/LPS	CD86, MHCII, iNOS [72]	TNFα, IL-6, IL-1b [74]	ROS secretion, microbial killing	pro-inflammatory
M2a	IL4/IL-13, glucocorticoids, immune complexes, and LPS	CD206, IL-1R, CCL17	TGF-β, IL-10, IGF, fibronectin	ECM deposition, angiogenesis, fibrosis	profibrotic
M2b	Role in wound healing is still subject to ongoing research [4,75]				
M2c	IL-10, TGF-β1	CD163, CCR1, TLR1, TLR8	IL-10, TGF-β	MMP secretion, ECM remodeling and deposition, angiogenesis, and apoptotic cell clearance	fibrolytic
M2d	Tumor-associated macrophages				

**Table 2:** A structured comparison of various *in vitro* and *in vivo* approaches targeting macrophage polarization.

Strategy	Mechanism	Evidence Level	Known Safety Profile	Optimal Timing	Clinical Feasibility
Apremilast [100]	inhibits NF-κB; shifts M1 → M2 polarization; promotes fibroblast migration	*in vitro* human macrophages; human epithelial and fibroblast cell lines	clinically approved drug with a generally well-characterized safety profile; perioperative safety in anastomotic healing remains unclear	Perioperative window; pre- or early postoperative	moderate—systemic oral therapy is clinically practical, but perioperative use requires further studies
MSC-derived secretome/fibrin glue [101]	MSC paracrine factors promote M2 polarization and cell proliferation; local delivery in fibrin glue	human/mouse cell lines *in vitro* and rat *in vivo*	Cell-free method, no side effects reported, fibrin glue already widely in use clinical practice	Intraoperative application at the anastomosis	moderate—local application is conceptually feasible, but clinical translation depends on standardized manufacturing and regulatory approval
HIF blockade [110]	Inhibition of HIF in macrophages leads to M2 polarization	murine *in vivo*; ischemic and septic colon anastomoses	Already in use in anemic patients with CKD	Perioperative window; pre- or early postoperative	low—attractive but unknown side effects and preclinical
Annexin-1 peptide Ac2-26 nanoparticles [111,112]	Inhibits NF-κB via formyl peptide receptor; promotes pro-resolving M2 response; delivered by oral pectin nanoparticles or intraoperatively	murine *in vivo*; DSS colitis and anastomotic models	Clinical safety has not yet been established; formulation-related safety and biodistribution require further evaluation	Perioperative window; pre- or early postoperative	low—promising preclinical concept, but clinical translation remains early
Injectable PGOT hydrogel [113]	Tissue-adhesive and antibacterial; directly reprograms macrophages toward an M2/CD206^+^ phenotype at the anastomosis	rat *in vivo*; colorectal anastomosis model	Preclinical biocompatibility appears promising, but long-term safety and local tissue effects require further validation	intraoperative application during anastomosis creation	low—attractive local-delivery strategy, but currently limited to preclinical development

## Data Availability

No new data were created or analyzed in this study. Data sharing is not applicable to this article.

## Abbreviations

The following abbreviations are used in this manuscript:

AH	Anastomotic healing
AL	Anastomotic leakage
ATP	Adenosine triphosphate
CCL2	C-C motif chemokine ligand 2
CKD	Chronic kidney disease
DAMP	Damage-associated molecular pattern
DC	Dendritic cell
DSS	Dextran sulfate sodium
ECM	Extracellular matrix
FcγR	Fc-gamma receptor
HMGP	High-mobility group protein
IBD	Inflammatory bowel disease
MCP-1	Monocyte chemoattractant protein-1
MIP-1α	Macrophage Inflammatory Protein-1
MMP	Matrix metalloprotease
MPS	Mononuclear phagocyte system
hucMSC	Human umbilical cord mesenchymal stem cell
PDE4	Phosphodiesterase-4
PGOT	injectable, antibacterial functional hydrogel
PPAR-γ	Peroxisome proliferator-activated receptor γ
PRR	Pattern recognition receptor
TIMP	Matrix metalloproteinase
Tip-DC	TNF- or iNOS producing dendritic cell
